# Identification of liver‐derived bone morphogenetic protein (BMP)‐9 as a potential new candidate for treatment of colorectal cancer

**DOI:** 10.1111/jcmm.17084

**Published:** 2021-11-28

**Authors:** Chen Cai, Timo Itzel, Haristi Gaitantzi, Carolina de la Torre, Emrullah Birgin, Johannes Betge, Norbert Gretz, Andreas Teufel, Nuh N. Rahbari, Matthias P. Ebert, Katja Breitkopf‐Heinlein

**Affiliations:** ^1^ Department of Medicine II University Medical Center Mannheim Medical Faculty Mannheim Heidelberg University Mannheim Germany; ^2^ Medical Research Center University Medical Center Mannheim Medical Faculty Mannheim Heidelberg University Mannheim Germany; ^3^ Department of Surgery University Medical Center Mannheim Medical Faculty Mannheim Heidelberg University Mannheim Germany

**Keywords:** bone morphogenetic protein‐9, colorectal cancer, ID1, noggin

## Abstract

Colorectal cancer (CRC) is a high‐incidence malignancy worldwide which still needs better therapy options. Therefore, the aim of the present study was to investigate the responses of normal or malignant human intestinal epithelium to bone morphogenetic protein (BMP)‐9 and to find out whether the application of BMP‐9 to patients with CRC or the enhancement of its synthesis in the liver could be useful strategies for new therapy approaches. In silico analyses of CRC patient cohorts (TCGA database) revealed that high expression of the BMP‐target gene ID1, especially in combination with low expression of the BMP‐inhibitor noggin, is significantly associated with better patient survival. Organoid lines were generated from human biopsies of colon cancer (T‐Orgs) and corresponding non‐malignant areas (N‐Orgs) of three patients. The N‐Orgs represented tumours belonging to three different consensus molecular subtypes (CMS) of CRC. Overall, BMP‐9 stimulation of organoids promoted an enrichment of tumour‐suppressive gene expression signatures, whereas the stimulation with noggin had the opposite effects. Furthermore, treatment of organoids with BMP‐9 induced ID1 expression (independently of high noggin levels), while treatment with noggin reduced ID1.

In summary, our data identify the ratio between ID1 and noggin as a new prognostic value for CRC patient outcome. We further show that by inducing ID1, BMP‐9 enhances this ratio, even in the presence of noggin. Thus, BMP‐9 is identified as a novel target for the development of improved anti‐cancer therapies of patients with CRC.

## 1 INTRODUCTION

Colorectal cancer (CRC) is a high‐incidence malignancy worldwide. Although comprehensive treatment strategies have helped to reduce mortality—especially in the case of early diagnosis—it still remains one of the deadliest cancer types. Therefore, new therapeutic targets for treatment of CRC are urgently needed.

Bone morphogenetic protein (BMP)‐9, a member of the TGF‐β family of cytokines that is constitutively produced by hepatic stellate cells of the liver, is a high‐affinity ligand of the type‐I receptor activin receptor‐like kinase1 (ALK1). BMP‐9 activates intracellular signalling through phosphorylation of Smads 1, 5 and 8 and induces expression of target genes like the inhibitors of differentiation (IDs). It is a strong enhancer of bone formation and plays an important role in regulating angiogenic processes and vascular homeostasis, and via its regulation of glucose metabolism and insulin resistance, it could serve as a potential therapeutic tool for treating diabetic disease.^1^, ^2^ In addition, we recently described that BMP‐9 enhances pro‐inflammatory responses of macrophages.^3^

Regarding its role in tumorigenesis and liver fibrosis, the results have been controversial. It seems that BMP‐9 can be both, a pro‐ as well as anti‐fibrogenic factor,^2^, ^4^, ^5^, ^6^, ^7^ and it can be pro‐ or anti‐proliferative to cancer cells.^8^, ^9^ Although the precise function of BMP‐9 in colorectal cancer remains unclear, a recent report by Fan et al. implied that high local BMP‐9 expression might be associated with poor outcome in patients with CRC.^10^ In this report, the authors investigated mRNA expression levels as well as protein presence of BMP‐9 in cancerous mucosa compared with normal mucosa, implying that in addition to the liver, the gut itself might be an important source for BMP‐9.

Three‐dimensional organoids generated from organ‐restricted adult human cells harbouring certain stem‐cell‐like properties represent a promising new research model that mimics the in vivo situation much better than conventional 2D set‐ups. In the future, such organoids should help to better understand the pathological mechanisms of disease, especially cancer, thereby allowing for the determination of possible new treatments. Successful generation of patient‐derived organoids was reported using tissue from several different organs, like stomach,^11^, ^12^ liver,^13^ pancreas^14^ and many others.^15^ The origin of most protocols used in this context lies in the initial work that described the generation of intestinal organoids derived from samples of human gut epithelium.^16^

Gut organoids are applied to study both homeostasis and cancerogenesis of gut epithelium, and they are promising tools for drug screening as well as personalized medicine approaches.^17^, ^18^

The aim of the present study was to investigate whether BMP‐9 could be a promising new target for therapy of patients with CRC. To do so, we first performed in silico analyses of patient data and complemented our findings with in vitro experiments using patient‐derived intestinal organoids. We directly compared normal (N) and cancerous (T) organoids derived from the same patient and studied the possible functions of BMP‐9 in normal as well as cancerous gut epithelium. We were able to define the ratio between expression of the BMP‐9‐target gene ID1 and the BMP‐inhibitor noggin as a new prognostic value in CRC. In contrast to the results of Fan et al.,^10^ our data strongly imply that by inducing ID1 expression BMP‐9 should be tumour‐suppressive in patients with CRC.

## 2 MATERIALS AND METHODS

### 2.1 Patient tissue samples

All samples were collected at University Hospital Mannheim. Tissue procurement was approved by the local Medical Ethics Committees (Reference No. 2014‐633N‐MA and 2016‐607N‐MA), and informed consent was obtained from all patients. We collected matched colon biopsies (cancer and non‐cancer areas) from the same patients. Human liver tissue for direct RNA preparation (see below) was derived from cancer‐distant areas of resection samples from patients with HCC or CRC liver metastases.

### 2.2 Mouse tissue samples

Five wild‐type, male C57BL/6 mice at the age of 4–5 months, with an average weight of 34 g, were used. Livers and colons were resected, washed with PBS, cut into small pieces and were immediately frozen in liquid nitrogen for later RNA purification (see below).

### 2.3 Generation of normal organoids (N‐Orgs)

Fresh human tissue biopsies (Table 1) were preserved in PBS on ice during transfer to the laboratory. Samples were washed immediately with PBS six times and cut into 2–4 mm^2^ pieces. The pieces were washed in Advanced Chelation Solution (ACS: 5.6 mM NA_2_HPO_4_, 8 mM KH_2_PO_4_, 96.2 mM NACL, 1.6 mM KCL, 43.4 mM sucrose and 54.9 mM D‐sorbitol) for another three times followed by a 1‐h incubation in ACS plus EDTA (40 ul of 2 mM EDTA in 10 ml ACS). At this point, the number of crypts in solution was counted: as long as it was less than 2 crypts/µl, the incubation time was extended. Crypts were centrifuged at 150 g for 10 min. Finally, 200 crypts per well of a 6‐well plate (non‐treated cell culture plates for suspension cultures) were combined with 100 µl matrigel and distributed evenly as drops within the well. The 6‐well plate was placed upside down in a 37°C, 5% CO_2_ incubator for 45 min. After that, the matrigel had solidified and was overlaid with 2 ml WENRA (Table 2) medium per well. The medium was exchanged every 48–72 h.

**TABLE 1:** Patient details

Patient ID	Gender	Age at time of biopsy (in years)	Tumour staging	Type of tumour
P080	f	65	T3, N1, M0	Rectum adenocarcinoma
P082	f	49	T3, N1, M0	Rectum adenocarcinoma
P090	m	74	T3, N0, Metachronous hepatic and Pulmonal metastases	Sigmoid adenocarcinoma

**TABLE 2:** List of media components

WENRA	Final concentration
Wnt3a	Conditioned medium, 50%
R‐Spondin	Conditioned medium, 20%
Noggin	Conditioned medium, 10%
B27	1:50
Nicotinamid	10 mM
NAC	1.25 mM
Primocin	100 mg/ml
EGF	50 ng/ml
Y‐27632	10 µM
A83‐01	500 nM
PGE2	10 nM
Gastrin	10 nM

Note

For initiation of organoid formation and for amplification of generated organoid lines, ENA and WENRAS media, containing many growth factors, inhibitors and other components as listed above, were used. ENA is equal to WENRA except that no Wnt3a or R‐Spondin is added. All components were combined in basal medium (=Advanced DMEM/F12). Conditioned medium was produced as previously described.^44^ To avoid interferences with the BMP pathway by any of these components, the medium was always changed to a basal medium, comprised of only advanced DMEM/F12 during the actual BMP‐9/noggin stimulations.

### 2.4 Generation of tumour organoids (T‐Orgs)

Human CRC biopsies (Table 1) were processed as described above for normal tissue, but the initial dissociation occurred in Liberase (13 U/ml dissolved in PBS) at 38°C for 1 h or until a single cell suspension was detected by microscopic observation. A total of 200,000 cells/well of a 6‐well plate were plated. For T‐Orgs, ENA (Table 2) medium was used and was exchanged every 48–72 h. Typical appearance of N‐ as well as T‐Orgs as documented by phase‐contrast microscopy is shown in Figure S1.

### 2.5 Freezing and thawing of organoids

Organoids grown on 6‐well plates were scraped of using a cell scraper. After centrifugation at 4°C, 800 g for 3 min, the cell pellet was dissolved in 90% FCS and 10% DMSO. Cryo‐tubes were preserved first in ‘Mr. Frosty’ containers for 24 h and then transferred into −80°C. For recovering, the organoids were shaken in a pre‐warmed 37°C water bath to thaw the freezing solution with the organoids as fast as possible. Organoids were then transferred into 10 ml DMEM containing 10% FCS, followed by centrifugation at 4°C, 800 g for 3 min. The pellet was dissolved in 100 µl matrigel and then plated again in 6‐well plates.

### 2.6 Isolation of RNA from cells, tissue or organoids

Cells were washed with PBS three times and then lysed in RNA lysis buffer (Peqlab). Frozen tissue samples were transferred to the lysis buffer and were disrupted by mechanical homogenization on ice. Matrigel drops containing organoids were scratched from the dish and centrifuged at 4°C, 800 g, for 3 min. Lysis buffer was added to the pellet containing the organoids, followed by gentle vortexing for 1 min. Then, total RNA was extracted using the peqGOLD Total RNA purification kit (Peqlab) according to the manufacturer's instructions.

### 2.7 Real‐time PCR

RNA was reverse transcribed into cDNA using the SensiFAST cDNA Synthesis Kit (Bioline, UK). Real‐time quantitative PCR (RT‐qPCR) was performed according to Analytik Jena innuMIX qPCR Master mix SyGreen protocol (Jena, Germany). Primers used for RT‐qPCR are listed in Table 3. mRNA expression was normalized using suitable housekeeping genes (HKG) as indicated in Table 3.

**TABLE 3:** Sequences of the primers used for real‐time PCR (5' => 3' orientation)

Target gene	Forward sequence	Reverse sequence
*hACVRL1*	CATCGCCTCAGACATGACCTC	GTTTGCCCTGTGTACCGAAGA
*hID1*	GTTTCAGCCAGTCGCCAGA	CAGCCGTTCATGTCGTAGAGCA
*hID3*	GAGAGGCACTCAGCTTAGCC	TCCTTTTGTCGTTGGAGATGAC
*hEng*	CGCCAACCACAACATGCAG	GCTCCACGAAGGATGCCAC
*h+mRS18*	CCATTCGAACGTCTGCCCTAT	TCACCCGTGGTCACCATG
*mGDF2*	CAGAACTGGGAACAAGCATCC	GCCGCTGAGGTTTAGGCTG
*mACVRL1*	GGCCTTTTGATGCTGTCG	ATGACCCCTGGCAGAATG
*mID1*	ATCCTGCAGCATGTAATCGAC	GCCTCAGCGACACAAGATG
*mB2 M*	TTCTGGTGCTTGTCTCACTGA	CAGTATGTTCGGCTTCCCATTC

Abbreviations

ACVRL1, ALK1, activin receptor‐like kinase1; B2 M, Beta‐2 microglobulin; GDF2, BMP‐9, bone morphogenetic protein 9; *h*, human; ID1, inhibitor of DNA binding 1 HLH protein; *m*, mouse.

Primers targeting human GDF2 (BMP‐9) were purchased from Qiagen (Germany): pre‐designed sequence: QT00210462; RS18, Ribosomal protein S18.

### 2.8 Microarray analyses

Total RNA was used for Affymetrix analyses only if the RIN values were above 7.8. cDNA was hybridized to Clariom S Human Microarrays (Affymetrix, Santa Clara, CA) at the Institute of Medical Research Center (Medical Faculty Mannheim). Raw data measured with the Affymetrix instrument were processed using the oligo package in R/Bioconductor. Background correction was performed by the Robust Multichip Average (RMA) algorithm. Application of quantile normalization was performed for the adjustment of distributions of expression levels between arrays. Differentially expressed genes (DEGs) were calculated with the limma package in the case of a single sample by subtracting the related reference. The differential expression profiles were used to perform a pre‐ranked gene set enrichment analysis (GSEA) based on the KEGG (Kyoto Encyclopedia of Genes and Genomes) subset of the canonical pathways provided by the molecular signatures database (MsigDB). A false discovery rate (FDR) lower than 0.25 and a p‐value lower than 0.05 were considered statistically significant. CMS subtypes were determined using the CMSCaller R package.^19^

### 2.9 Bioinformation analyses

The TCGA‐COAD+READ cohorts were used to analyse the relationship between patients’ overall survival times and the expression levels of ID1 or noggin, respectively, by GEPIA (http://gepia.cancer‐pku.cin).

### 2.10 Statistics

GraphPad Prism 8.0a was used for statistical analyses (GraphPad Software Inc.). For statistical differences between groups, we used paired two‐sample *t*‐tests. Error bars are standard deviation, and *p* < 0.05 was considered statistically significant (*).

## 3 RESULTS

### 3.1 High expression of the BMP‐target gene ID1 as well as low levels of the BMP‐inhibitor noggin correlates with better survival of patients with CRC

**FIGURE 1:**
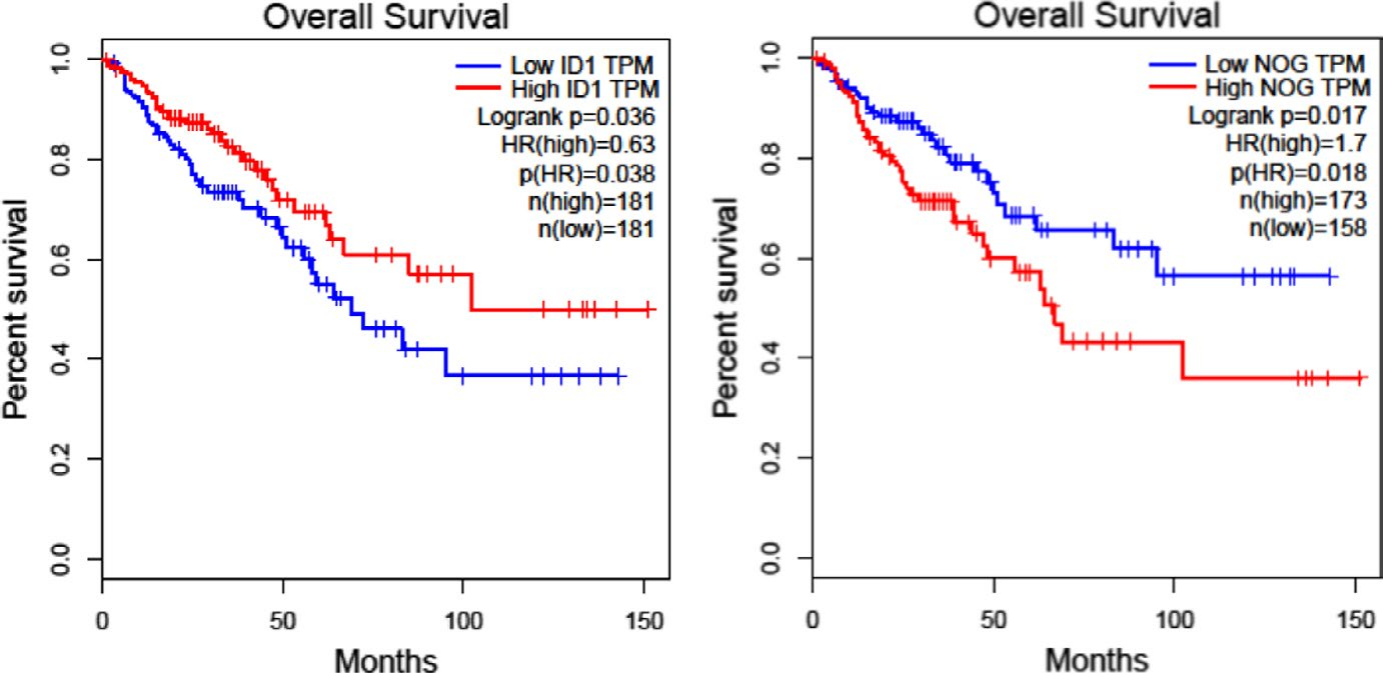
In silico analyses on the correlation between BMP‐pathway activity and patient survival in colorectal cancer (CRC). Graphical depictions of the prognostic value of the mRNA levels of ID1 (left graph) and noggin (right graph) in samples from patients with CRC. Data from the TCGA‐COAD+READ cohorts (colon and rectum adenocarcinoma) were analysed using GEPIA. High levels of ID1 expression show a significant correlation (*p* = 0.038) with longer survival times, whereas high noggin expression has the opposite effect (*p* = 0.018)

The first aim of this study was to investigate whether the BMP pathway plays a role in CRC. One prototypic target gene induced by BMPs is *inhibitor of differentiation* (ID)‐1. Noggin, a modulator of BMP signalling belonging to the group of BMP antagonists,^20^ was reported to correlate with reduced survival of patients with gastric cancer.^21^ We took ID1 expression as an indicator for active BMP signalling and noggin for inhibited signalling and analysed patient data from the Cancer Genome Atlas (TCGA; cohorts colon adenocarcinoma (COAD) plus rectum adenocarcinoma (READ)). We correlated ID1 or noggin expression, respectively, with the survival times of the corresponding patients. The resulting curves indicate that high ID1 expression significantly correlates with longer survival, whereas high noggin expression was significantly associated with decreased survival (Figure 1).

### 3.2 Expression of BMP‐9 and its receptor, ALK1, in non‐malignant colon versus liver tissue

Since ID1 is induced by many BMPs—including BMP‐9—but noggin should not inhibit BMP‐9,^22^ we next addressed whether BMP‐9 signalling could play a beneficial role in CRC development by upregulating ID1.

**FIGURE 2:**
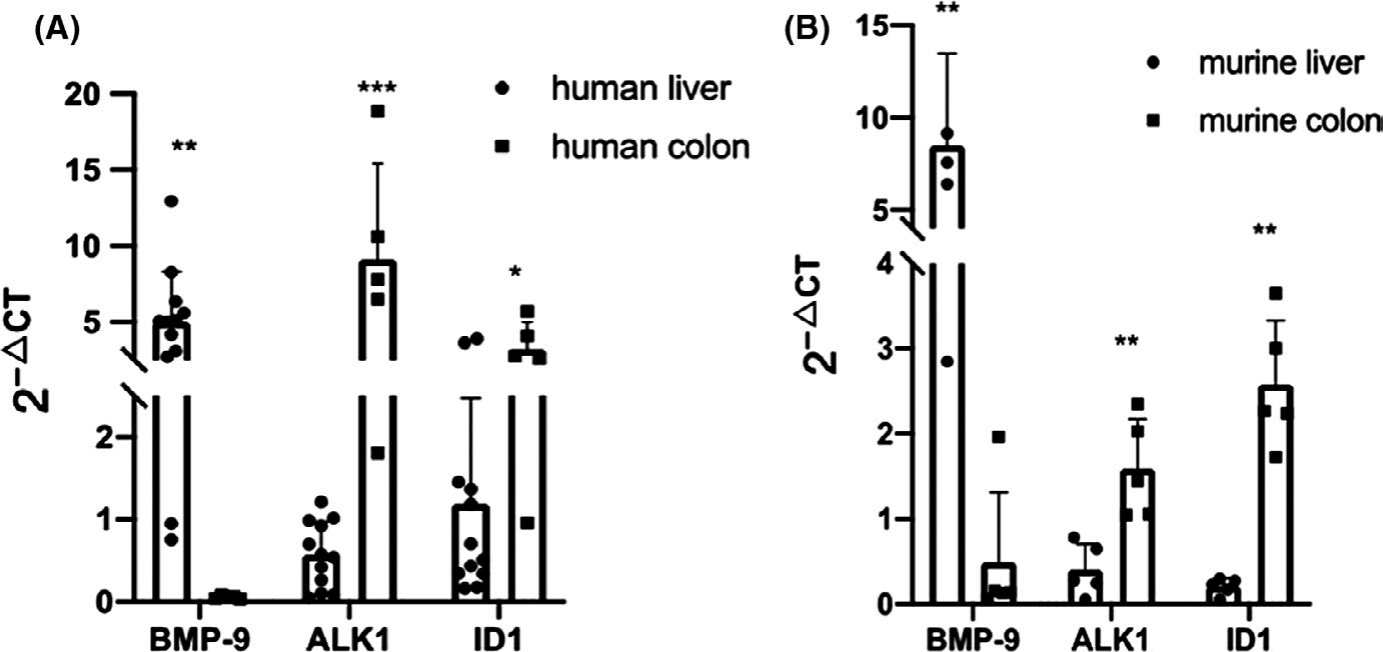
BMP‐9 is mainly expressed by liver, while ALK1 and ID1 are mainly expressed by colon. (A) 12 human liver samples and 6 human colon samples (both from non‐malignant areas) were collected and processed for RT‐PCR. CT values for BMP‐9, ALK1 and ID1 were normalized to the house‐keeping gene rS18. (B) 4 mouse liver samples and 5 mouse colon samples were obtained from healthy mice and processed for RT‐PCR analyses. CT values for BMP‐9, ALK1 and ID1 were normalized to the house‐keeping gene b2M. Statistically significant changes are indicated as follows: *p > 0.05; **p > 0.01; ***p > 0.001

We, therefore, first analysed the basal expression levels of BMP‐9, its specific receptor ALK1 and ID1 in non‐malignant tissue samples from human as well as murine colon and compared it with the liver (Figure 2). As expected, healthy liver expresses much higher levels of BMP‐9 than colon tissue. In contrast, ALK1 and ID1 were much stronger expressed in the colon, suggesting that the gut epithelium should generally be responsive to BMP‐9 stimulation.

These results support the hypothesis that liver‐derived BMP‐9, reaching the gut via the bloodstream, can control ID1 expression in the gut epithelium.

### 3.3 Generation and characterization of epithelial organoids derived from normal and cancerous biopsies of individual patients

To further address the hypothesis that BMP‐9 might affect CRC progression by regulating ID1 expression and that noggin exerts the opposite effect, we generated organoids from human tissue samples (biopsies) of 3 patients with CRC according to the previously published protocols.^16^ We directly compared organoids derived from normal tissue (N‐Orgs) with those from cancerous samples (T‐Orgs) of the same individual patients. We then stimulated these organoids in vitro with BMP‐9, noggin or a combination of both (see scheme in Figure S2).

Since this method of organoid generation is based on the isolation of gut epithelial cells with stem‐like properties, we can expect that the cell types that are present in the organoids should all be progeny from these cells and that non‐epithelial cells should be absent. To prove this assumption, we performed microarray analyses of all samples and investigated the expression levels of different cell‐type markers. Indeed, the results show that fibroblast‐ as well as endothelial cell markers were barely expressed (Figure S3). The expression levels of the different epithelial cell‐type markers were not profoundly different between normal and tumour‐derived organoids, supporting the assumption that despite the accumulation of mutations in the tumour, both types of organoids derive from epithelial cells that are still similarly able to differentiate.

We then used the expression data from the 12 T‐Org samples and analysed them using ‘CMScaller’, an R package that was established to define the consensus molecular subtypes (CMS) of samples from pre‐clinical models of colorectal cancer (like organoids) based on their expression profiles.^19^ The results, presented in Figure S4A, imply that P080 belongs to CMS1 (with high p‐values) and P082 probably belongs to CMS3 (intermediate p‐values). For P090, the calculation grouped this patient into either CMS2 or 4, though with very low statistical power. These low p‐values are due to the low input: CMScaller is described to only deliver results of high statistical power if data from *n* > 40 are used. Nevertheless, the data from our 12 samples suggest that P090 is not of CMS 1 or 3. We further noted that patient 090 seems to have mutations in the genes for APC (Wnt signalling) and TP53, a tumour suppressor. Looking at the expression levels of these two genes, we found a strong down‐regulation in patient 090 in contrast to both other patients (Figure S4B). The low expression of p53 can be assumed to enhance tumour progression.^23^ Our finding that P090 does not belong to CMS 1 or 3 fits well with published data, showing that TP53 mutation frequency is highest in CMS 2 and 4.^24^ Smeby et al. also showed that the consequences in terms of expression changes due to TP53 mutation are highest in CMS1 and 4 (not in 2); in CMS1, it leads to reduced T‐cell activity, and in CMS4, it leads to enhanced proliferation. Our P090 has enhanced expression of the proliferative gene PCNA and reduced expression of the anti‐proliferative CDKN1A (Figure S4B), suggesting that P090 possibly belongs to CMS4.

When further analysing groups of genes that were changed in T‐Orgs compared with N‐Orgs, we found, as expected, enhanced expression of genes related to cell growth and metabolism, whereas genes related to glycan biosynthesis and immune regulation were decreased in T‐Orgs (Figure S5).

When directly comparing expression changes within the individual patients, we found strong patient‐specific expression patterns (Figure S6). This is not surprising, since the tumours of these three patients seem to belong to 3 different groups of CMS. Among the top up‐regulated and down‐regulated genes, only few were equally regulated in all three patients. Among them was protein tyrosine phosphatase, receptor‐type O (PTPRO), a protein that belongs to the R3 subtype family of receptor‐type protein tyrosine phosphatases. PTPRO has been suggested as a tumour suppressor in CRC,^25^ and it was clearly up‐regulated in the T‐Orgs of all three patients compared with their corresponding N‐Orgs (Figure S6B). There was no single common gene among the top 30 down‐regulated genes in all three patients (Figure S6A).

### 3.4 Opposite effects of BMP‐9 and noggin, respectively, on N‐ and T‐organoids

According to our in silico analyses (Figure 1), BMP‐9‐mediated induction of ID1 should have tumour‐suppressive effects, while noggin should cause tumour‐promoting changes. To further address this theory in vitro, we stimulated the organoids with BMP‐9, noggin or a combination of both (see scheme in Figure S2) and analysed the resulting expression profiles.

**FIGURE 3:**
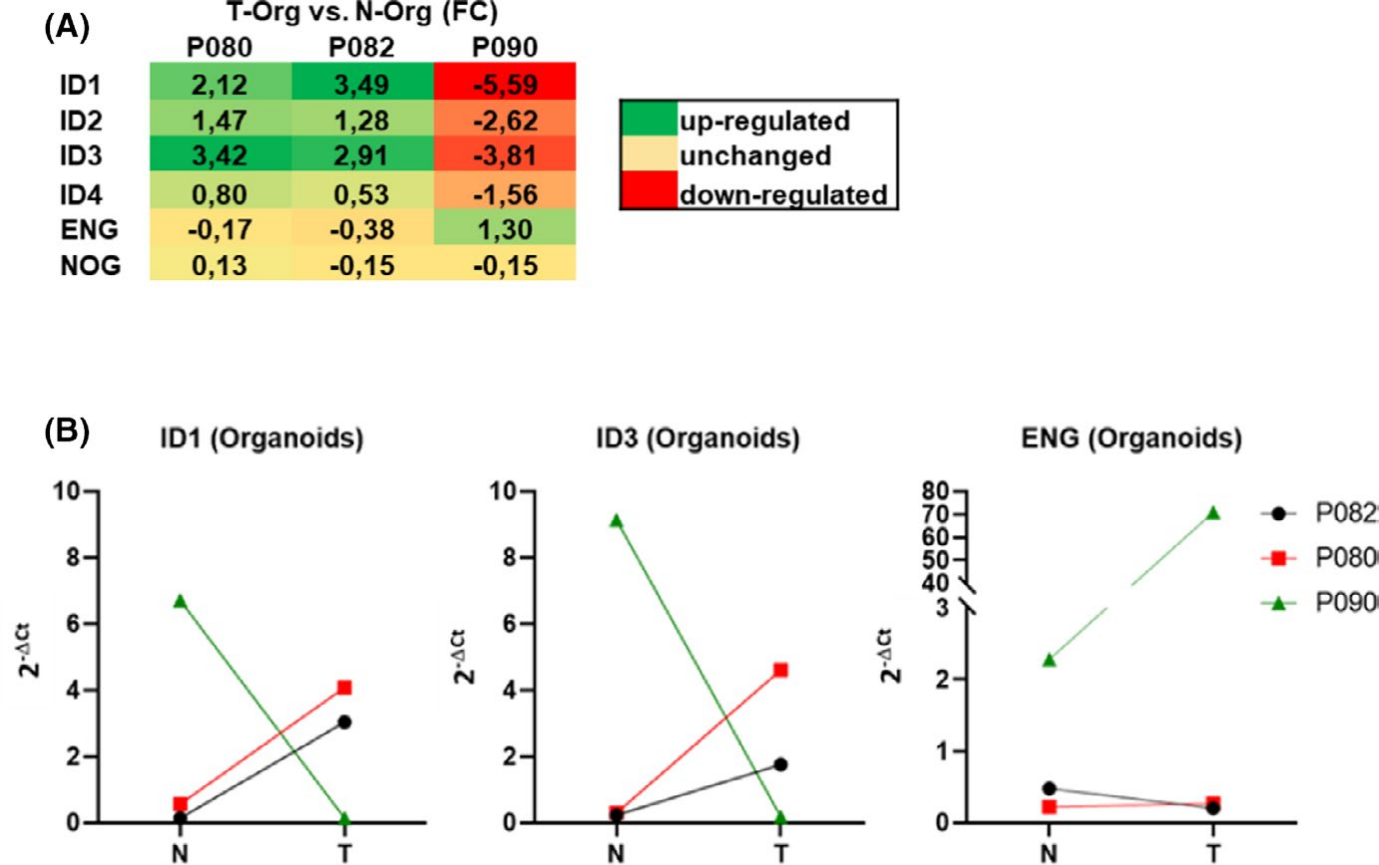
Analyses on the expression changes of the BMP‐target genes *inhibitor of differentiation* (ID) 1–4 in T‐Orgs derived from 3 colorectal cancer patients. (A) Expression changes of ID‐1 to ‐4, the BMP‐co‐receptor endoglin (ENG) and the BMP‐inhibitor noggin (NOG) in T‐Orgs compared with their corresponding N‐Orgs are shown. Up‐regulated expression is shown in green and down‐regulated in red (unchanged = yellow). Individual fold changes (LogFC) of each patient as calculated from array analyses are given. (B) Verification of the array data by regular real‐time PCR: Expression changes for ID1, ID3 and ENG closely matched the corresponding array data

Focussing first on the comparison between basal expressions in N‐ versus T‐Orgs of the individual patients, we found a strong down‐regulation of ID1‐4, specifically in the T‐Orgs of patient 090. In the other two patients’ organoids, these genes were up‐regulated in T‐Orgs. At the same time, the BMP co‐receptor, endoglin (ENG), was oppositely regulated, while noggin was mainly unchanged (Figure 3A). The expression changes of ID1, ID3 and endoglin (ENG) in T‐Orgs compared with their corresponding N‐Orgs were additionally confirmed by regular real‐time PCR (Figure 3B). In line with the concept that ID1 expression is associated with better patient outcome, patient 090—having strongly down‐regulated expression of ID1 in the T‐Orgs—showed a rather bad outcome, with the development of liver and lung metastases within two years after surgery (Table 1).

**FIGURE 4:**
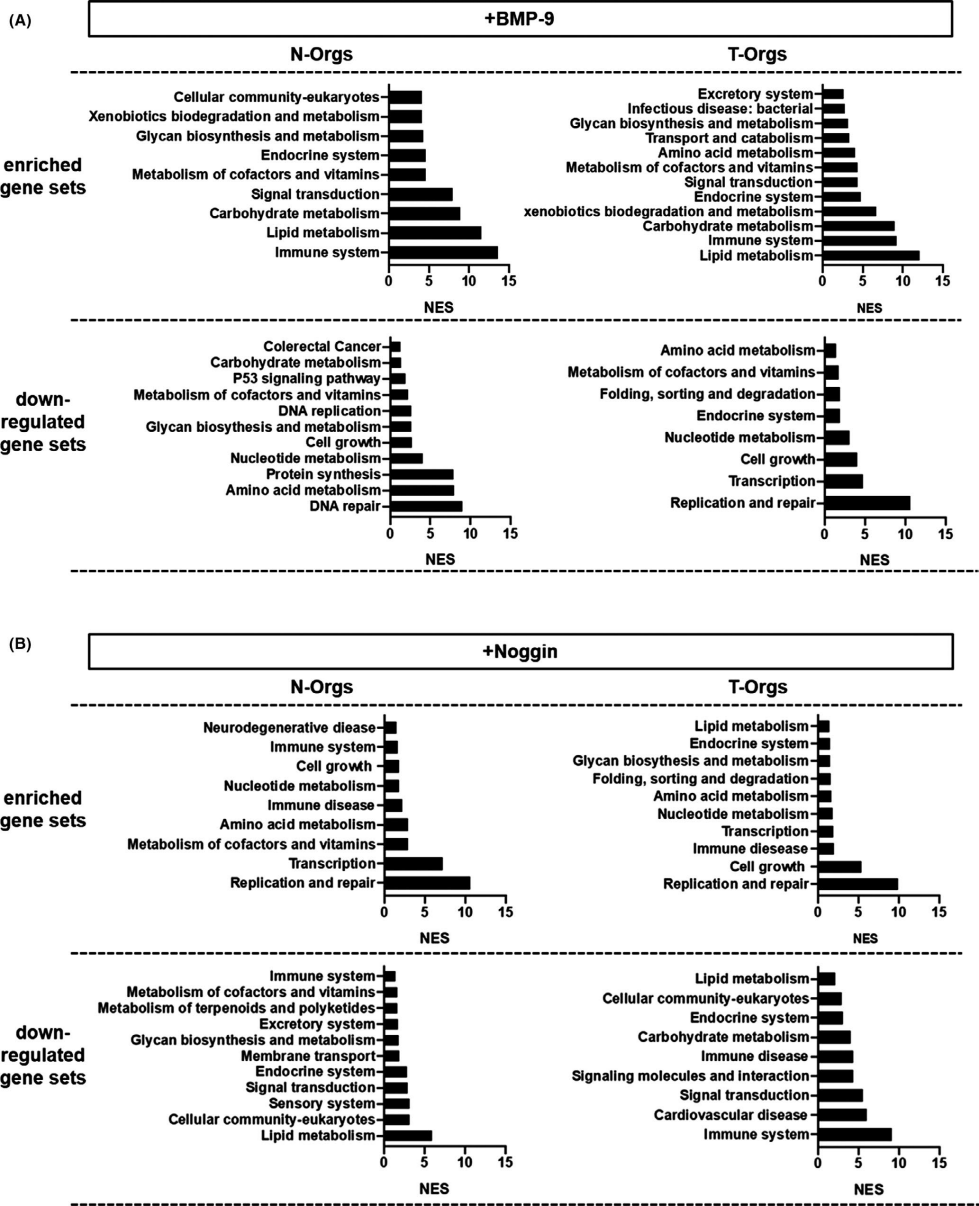
BMP‐9‐ and noggin‐responses of N‐ and T‐organoids. After stimulation of organoids with (A) BMP‐9 (5 ng/ml) or (B) noggin (100 ng/ml) for 48 h, gene sets enrichment analyses (GSEA) were performed focusing on the Kyoto Encyclopedia of Genes and Genomes (KEGG). A false discovery rate (FDR) lower than 0.25 and a *p*‐value lower than 0.05 were considered as statistically significant. NES: normalized enrichment score

Enrichment analyses were performed to analyse groups of genes that were enriched or reduced by stimulation with either BMP‐9 or noggin in N‐ and T‐Orgs (Figure 4). Among other effects, BMP‐9 seems to antagonize, while noggin seems to enhance gene expressions related to cell growth. In addition, BMP‐9 enhanced and noggin down‐regulated genes related to immune response. Interestingly, these effects are similar in N‐ and T‐Orgs (Figure 4).

### 3.5 BMP‐9 induces while noggin down‐regulates ID1 expression in N‐ as well as T‐organoids; therefore, BMP‐9 supports a high ratio of ID1/noggin, which predicts better patient survival

**FIGURE 5:**
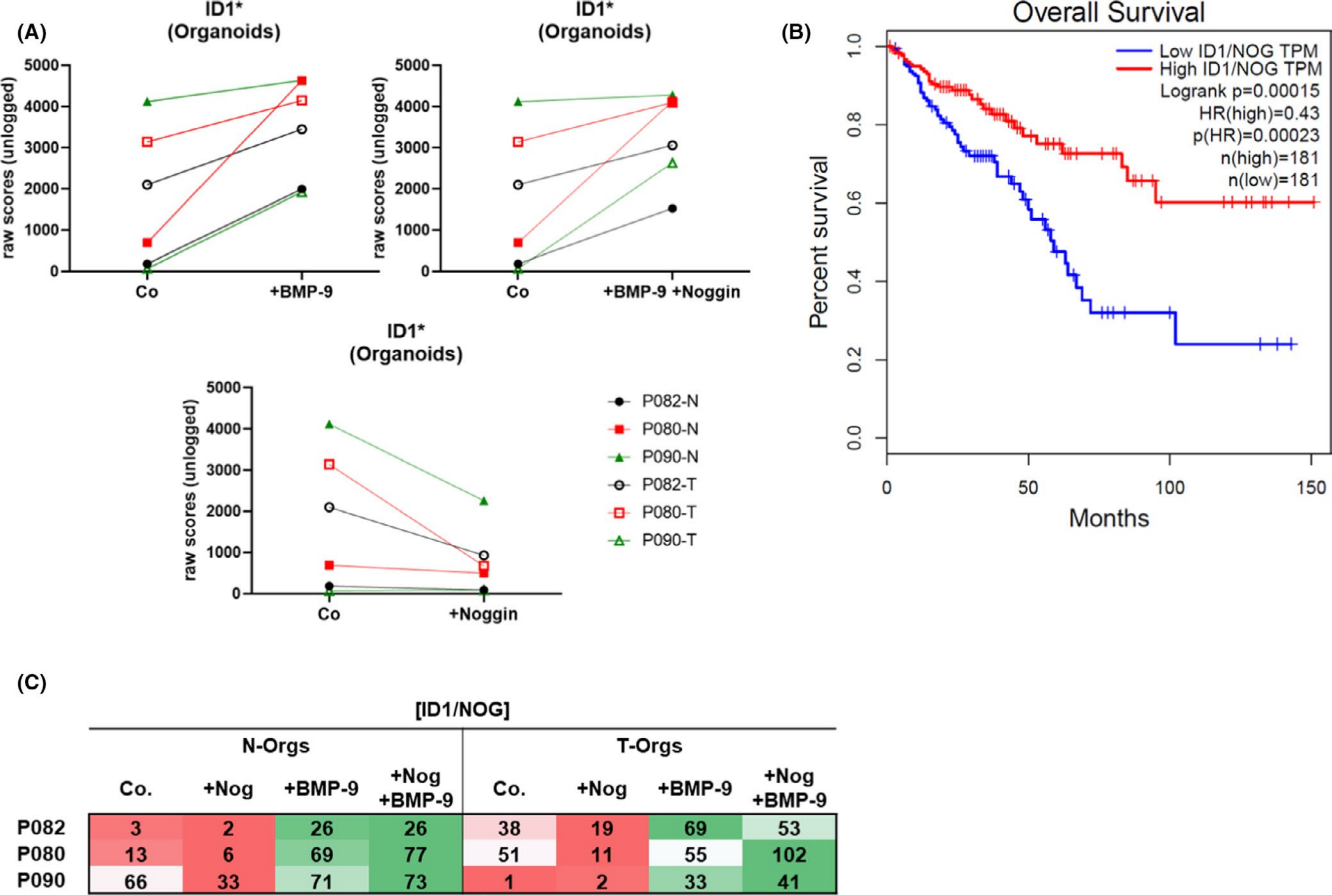
Noggin reduces and BMP‐9 enhances ID1 expression in organoids and high ID1 in combination with low noggin expression significantly correlates with better survival of CRC patients. (A) Organoids were derived from human biopsies of normal and cancerous gut mucosa as depicted in Figure 1 followed by in vitro stimulation with either recombinant noggin (100 ng/ml) or BMP‐9 (5 ng/ml) or both together. After 72 h, RNA was isolated and processed for Affymetrix array analyses. The raw scores (unlogged values) for the individual ID1 expression in each sample (N as well as T of each patients organoids) are plotted, and values of untreated (Co.) are compared with either BMP‐9‐treated (+BMP‐9), noggin‐treated (+Noggin) or treated with both (+BMP‐9 +Noggin). For statistics, all 6 controls were compared with all 6 treated samples and significance was calculated using the paired *t*‐test. *p > 0.05. (B) Graphic depictions of the prognostic values of the mRNA levels of the ID1‐to‐noggin ration in CRC patients from the TCGA‐COAD+READ cohort analysed by GEPIA. High ID1/NOG expression levels significantly correlate with better survival of the patients. (C) Calculation of the ratio between ID1 and NOG in each patient's organoid sample using the array data depicted in A. green, enhanced ratio; red, reduced ratio

As described earlier, for patients with gastric cancer, high expression of noggin is significantly associated with reduced patient survival time,^21^ and noggin was described to inhibit BMP signalling in some contexts.^22^ Since we now found that high ID1 correlates with better patient survival (Figure 1) and because we wanted to prove that noggin does not inhibit BMP‐9 signalling in our model, we investigated the direct effects of noggin, BMP‐9 and the combination of both on ID1 expression in N‐ as well as T‐Orgs of all 3 patients. In all organoids except T‐Orgs from patient 090, noggin led to reduced expression of ID1 as determined by microarray analyses. BMP‐9, in turn, induced ID1 expression in all organoids, including the T‐Orgs of patient 090. Additionally, this inducing effect of BMP‐9 was not inhibited by noggin (Figure 5A). We confirmed these findings using regular real‐time PCR (Figure S7). Similar tendencies of regulation were also found for IDs 2 and 3 (Figure S8).

As shown in Figure 1, we observed a significantly better rate of patient survival with high expression of ID1 or low expression of noggin. To get further insight about the regulation of ID1 expression in the organoids of the 3 patients, we analysed the basal expression levels of a selection of TGF‐β/BMP‐ligands and ‐signalling pathway components (Figure S9). The data show highest expression of BMP‐4, ALK3 and SMAD5. Therefore, we assume that basal expression of ID1 in the gut epithelium (normal as well as cancerous) is endogenously induced by this BMP‐4/ALK3 pathway, which can be expected to be inhibited by noggin.^26^

If this is true, high noggin levels should antagonize ID1 expression, and the combination of high ID1 with low levels of noggin should support even better survival than high ID1 alone. We tested this hypothesis again using in silico data from TCGA and found that a high value for the ratio between ID1 and noggin is very significantly (*p* = 0.00023) correlated with better patient survival (Figure 5B). These data demonstrate that the ratio between ID1 and noggin is an important new predictor of patient outcome in CRC.

To further support our hypothesis that BMP‐9 potentially exerts beneficial effects by inducing ID1, we next calculated how BMP‐9 or noggin change the ID1/NOG ratio in the three patient's organoids. The result is shown in Figure 5C and confirms that BMP‐9 always led to an enhanced ID1/NOG ratio (and this BMP‐9 effect was not inhibited by adding noggin in parallel), while noggin itself always reduced the ratio. The T‐Orgs from P090 had a very low basal level of this ratio, but also, application of BMP‐9 was able to enhance it, thus supporting our conclusion that BMP‐9 could be a promising factor for new therapy approaches against CRC.

## 4 DISCUSSION

In contrast to most other members of the TGF‐β family of cytokines, BMP‐9 is constitutively expressed and constantly circulates in the bloodstream of healthy individuals in an active conformation.^27^ Our previous work, as well as the work of others, has demonstrated that the liver is the main site of BMP‐9 production in the body.^5^, ^28^ However, expression of BMP‐9 by colon cells has also been reported.^29^ ALK1, the type‐I receptor with highest affinity to BMP‐9, was described to be expressed mainly in endothelial cells,^30^, ^31^ including liver sinusoidal endothelial cells,^3^ whereas normal hepatocytes seem to express mainly the related type‐I receptor ALK2.^5^ Nevertheless, in human liver cancer (HCC), ALK1 mRNA expression was significantly up‐regulated.^5^, ^32^ Whether BMP‐9 might be a promising candidate for new therapy approaches to treat patients with CRC was not known so far.

We performed in silico analyses on a cohort of human samples from colon and rectum adenocarcinoma compared with normal colon tissue and found that high expression of the BMP‐target gene ID1 is positively correlated with longer survival (Figure 1). Up to now, increased expression of ID proteins has mostly been associated with tumour progression in several types of cancer.^33^ Beneficial effects of ID1 in CRC progression would also be in contrast to findings in prostate cancer, where increased expression was reported to be associated with poor survival of the patients.^34^ Our data indicate that this is different in CRC. In the gut, Zhang et al.^35^ have demonstrated that ID1 is not only associated with stemness of cells from the crypt base, but also that its expression is needed for efficient regeneration upon challenge with colitis‐inducing dextran sodium sulphate (DSS) in mice. This implies that ID1 might be an important factor in maintenance and regaining of epithelial functionality of the gut mucosa. Such action of ID1 might very well act tumour‐suppressive and protective in vivo.

Noggin, a modulator of BMP signalling, was reported to correlate with reduced survival of patients with gastric cancer,^21^ and our data reproduce this observation now for patients with CRC. Noggin belongs to the group of BMP antagonists, and it inhibits signalling of, for example, BMPs‐2 and 4, but not that of BMPs‐3, 6, 9, 10 or 15.^20^, ^22^

When comparing the basal expression of BMP‐9 in the normal human liver with normal human colon epithelium, we found that although BMP‐9 is produced by hepatic stellate cells, and not by hepatocytes, whole liver lysates still express about 50‐fold higher levels of BMP‐9 than gut epithelium (Figure 2). Oppositely, expression of the specific BMP‐9 receptor ALK1 was much higher in the gut, and the finding that the BMP‐target gene ID1 was also much higher in gut supports the concept that there is active BMP signalling in the normal gut. How much of this signalling might be initiated by liver‐derived or locally produced BMP‐9, or rather by other BMPs like BMP‐4, remains to be addressed. However, the presence of ALK1 implies that intestinal cells should generally be responsive to BMP‐9.

In order to further investigate the effects of BMP‐9 and noggin on the normal gut mucosa as well as on colorectal cancer cells, we searched for a human in vitro model system. In recent years, work from the Clevers’ lab especially has demonstrated that adult human stem cells from the colonic crypts can form 3‐dimensional cell culture models (organoids) in vitro that preserve many of the functions and phenotypes of the original human gut epithelium.^36^ The fact that stem cells from healthy epithelium are constantly renewing the gut mucosa throughout the lifetime represents the basis for the in vitro formation of gut organoids.^37^ In colon cancer, accumulation of several mutations within such cells typically leads to uncontrolled proliferation, but these mutated stem cells can still give rise to organoids in vitro.^16^ In the present study, we analysed the gene expression changes from normal to cancer‐forming epithelium, as well as its responses to BMP‐9 or noggin stimulation, using organoids derived from normal (N‐Org) as well as cancerous gut epithelium (T‐Org) obtained from biopsies of three individual patients. We characterized the T‐Orgs and used them as models representing 3 different consensus molecular subtypes (CMS): CMS1, 3 and 4 (Figure S4).

The overall expression levels of the different cell‐type markers were not profoundly different between normal and tumour‐derived organoids, supporting the assumption that both are derived from the same type of epithelial cell. A previously published analysis using normal versus CRC organoids focussed on changes on the protein level and reported strong patient‐specific features.^38^ This is not surprising when considering the fact that CRCs can be subdivided into at least 4 CMS. Also, in our 3 lines, the changes in gene expression patterns that we found in comparing T‐Orgs with N‐Orgs were highly patient‐specific. Nevertheless, one gene, PTPRO, was among the top 50 up‐regulated genes in all three patients. Gene expression analyses of 688 primary colon tumour samples revealed that PTPRO mRNA expression is strongly down‐regulated in patients with colon cancer with a poor prognosis,^39^ and loss of PTPRO expression is associated with increased resistance to EGFR inhibition,^25^ suggesting that increased expression of PTPRO might be beneficial. It remains to be investigated whether PTPRO levels in T‐Orgs are of equal diagnostic relevance as compared to the original tumour.

We discovered an interesting relationship between noggin and ID1. While treatment of T‐Orgs with noggin down‐regulated ID1, BMP‐9 up‐regulated ID1 (as expected) and was not inhibited by noggin (Figure 5A). We further found that the ratio between noggin and ID1 expression was significantly correlated with patient survival (Figure 5B). This means that high noggin expression, in combination with low ID1, is a marker for bad prognosis. Therefore, noggin is understood to be tumour‐promoting through its reduction in ID1 expression, while BMP‐9 is understood to be tumour‐suppressive in how it shifts the ID1/NOG ratio in a favourable direction. In this context, it is promising to see that despite the strongly patient‐specific responses of the organoid lines, the BMP‐9 response was uniformly strong in all. Unexpectedly however, the combination of noggin plus BMP‐9 caused an even further improved ratio of ID1/NOG in 5 of the 6 organoid lines (Figure 5C). We initially chose this combined treatment condition to demonstrate that noggin does not inhibit BMP‐9 signalling, which indeed was the case. The mechanistic reason for this synergistic effect of noggin plus BMP‐9 remains to be investigated.

The microarray analyses further indicate that BMP‐9 positively regulates genes involved in responses of the immune system, as well as lipid and carbohydrate metabolism. Cell replication and growth were, in turn, antagonized by BMP‐9. Noggin, on the contrary, induced rather opposite effects to BMP‐9. Interestingly, these functions seem to be more or less conserved in the T‐Orgs, implying that the effects of BMP‐9 (as well as noggin) should remain similar in normal as well as tumour epithelium. Further supporting the beneficial role of BMP signalling, the BMP pathway was abrogated in a large number of sporadic cases of CRC,^40^ and BMP pathway suppression was described to be associated with neoplastic changes in the colon of uPA‐deficient mice.^41^

Regarding BMP signalling in the individual organoids, it is interesting to note that there was a down‐regulation of the BMP/Smad‐target genes ID1 and ID3 specifically in the T‐Orgs of patient 090. In the other two patients, these genes were clearly up‐regulated in the T‐Orgs compared with the corresponding N‐Orgs. At the same time, the BMP co‐receptor, endoglin (ENG), was oppositely regulated. High expression of endoglin (especially on CAFs) has recently been connected to enhanced invasion and aggressiveness of CRC with the use of patient‐derived tissue samples.^42^ In line with this, patient 090 especially exerted a rather aggressive further course of disease and developed distant metastases in the liver and lung within two years after time of biopsy. We also found that the organoids of P090 most likely harbour mutations in the driver genes APC and p53, resulting in very low expression of these genes. This is in line with the aggressive course of disease of P090, as well as published results from a different cohort.^23^ Interestingly, a correlation between BMP‐9 and p53 has been described earlier by Li et al.^43^: in a human CRC cell line (HCT116), BMP‐9 promotes p53 activity and thereby reduces tumour growth in a xenotransplantation mouse model.

In summary, we present the ratio between ID1 and noggin expression as a new marker for prognosis of patients with CRC, and our data strongly imply that application of BMP‐9 or induction of its expression in the liver should be a promising new strategy for future therapy approaches against initiation and progression of CRC in patients.

## CONFLICTS OF INTEREST

The authors confirm that there are no conflicts of interest.

## AUTHOR CONTRIBUTIONS

**Chen Cai:** Conceptualization (supporting); Data curation (equal); Formal analysis (equal); Investigation (lead); Methodology (lead); Software (equal); Writing – original draft (equal); Writing – review & editing (equal). **Timo Itzel:** Data curation (equal); Software (equal). **Haristi Gaitantzi:** Methodology (supporting). **Carolina de latorre:** Data curation (supporting). **Emrullah Birgin:** Validation (supporting). **Johnannes Betge:** Visualization (supporting). **Norbert Gretz:** Validation (supporting). **Andreas Teufel:** Supervision (supporting). **Nuh Rahbari:** Supervision (supporting). **Matthias Ebert:** Resources (supporting); Supervision (supporting). **Katja Breikopf‐Heinlein:** Conceptualization (lead); Data curation (equal); Resources (lead); Supervision (lead); Writing – original draft (equal); Writing – review & editing (equal).

## Supporting information

Figure S1‐S8
