# Supplementary material for: Identification of liver‐derived bone morphogenetic protein (BMP)‐9 as a potential new candidate for treatment of colorectal cancer
Source: J Cell Mol Med. 2021 Nov 28;26(2):343–53. doi: 10.1111/jcmm.17084 (PMC8743662; doi:10.1111/jcmm.17084)
Supplement: Supplementary file 1 — Figure S1‐S8 [file JCMM-26-343-s001.pdf]

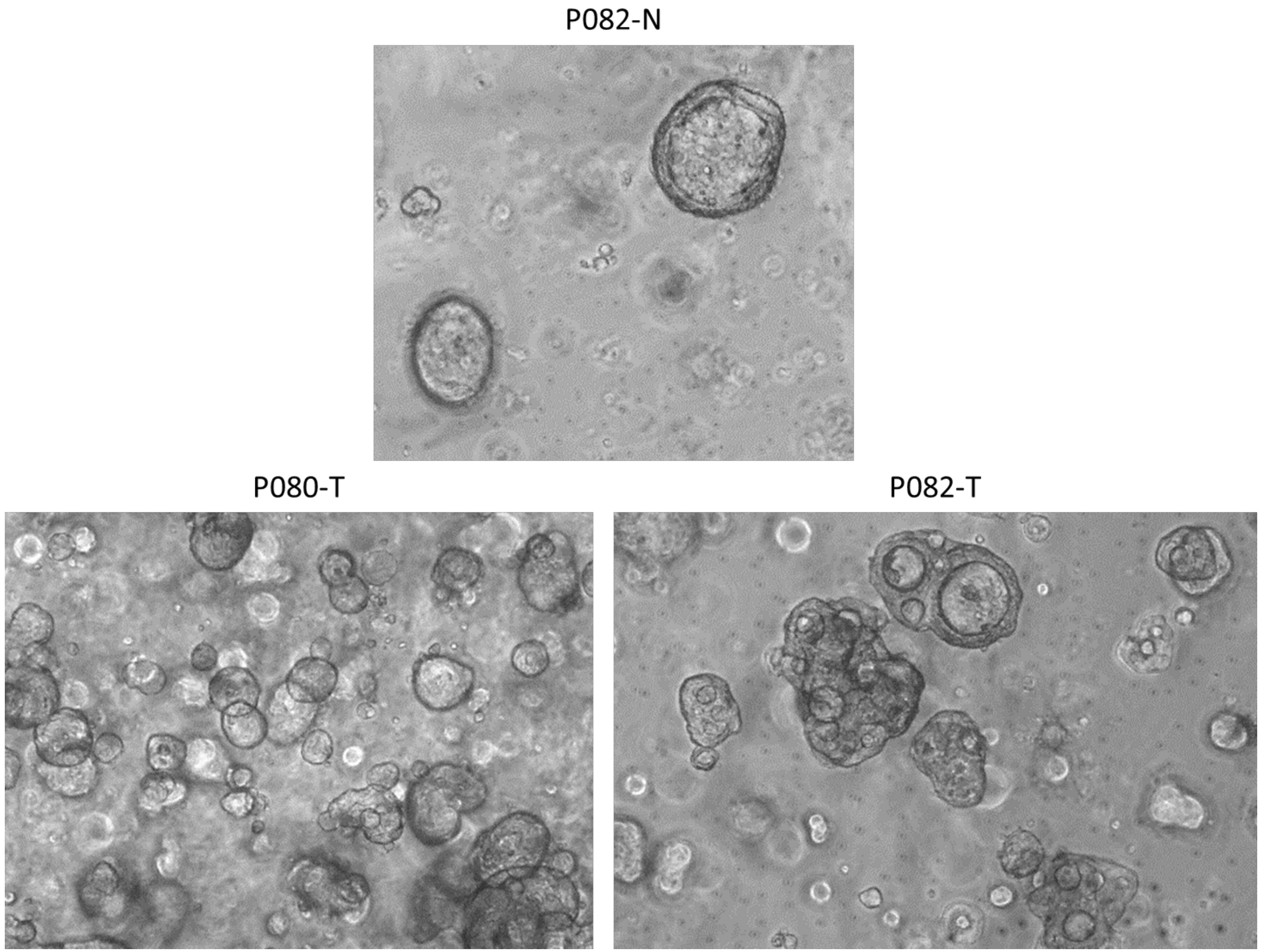

Suppl. Fig. 1: Morphology of N- as well as T-Organoids in advanced medium. 3 days after changing the medium to Advanced medium, morphology of N- as well as T-organoids was observed under phase-contrast microscopy. Magnification 40X.

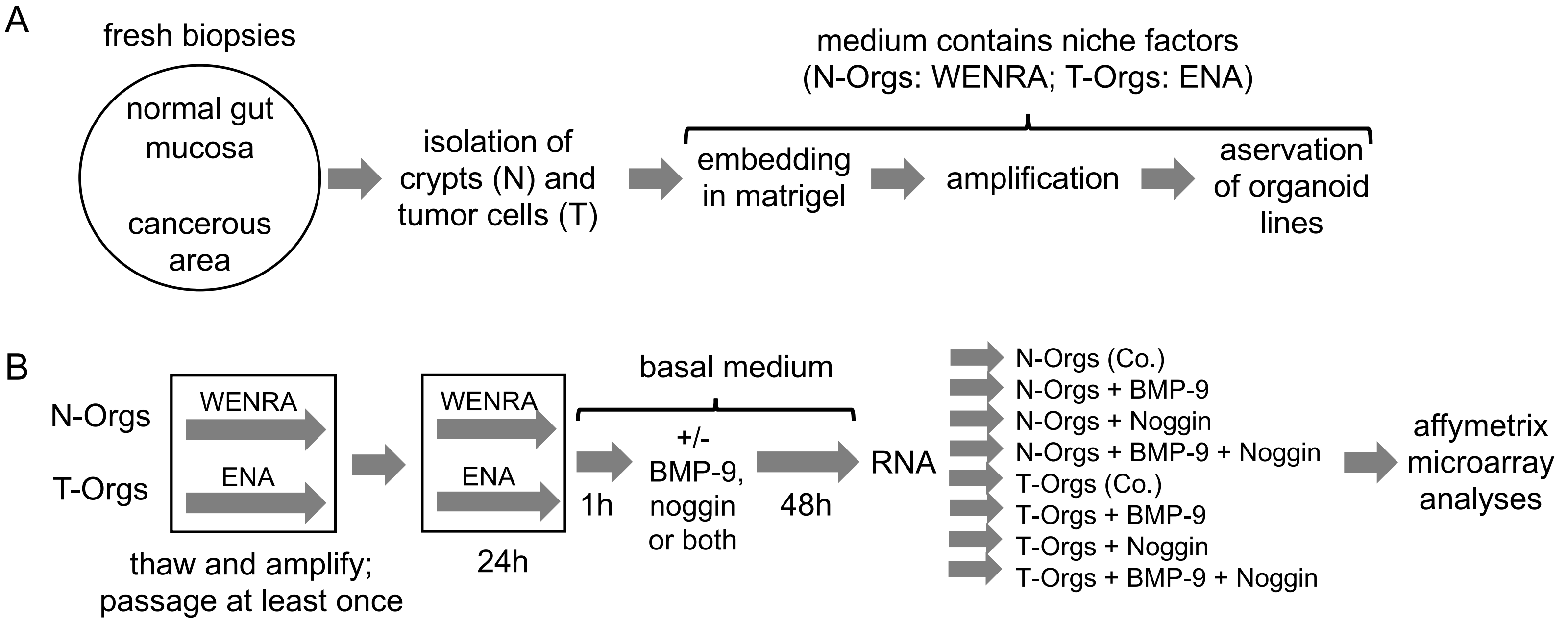

Suppl. Fig. 2: Schematic presentation of the experimental work-flow. A) Generation of organoids from normal (N) and cancerous (T) areas using colon biopsies from the same patient. For generation of optimal growth conditions, the organoids were cultured in medium containing defined factors of the stem cell niche of the colon crypts (WENRA for N-Orgs and ENA for T-Orgs; see materials and methods section for exact composition). B) After thawing the organoid lines, they were first amplified and after at least one passaging they were cultured for 24h in their corresponding amplification media and one hour before stimulation with BMP-9 (5 ng/ml) or noggin (100 ng/ml) or both, the medium was changed to a basal medium (see materials and methods for details). After two further days total RNA was isolated and processed for Affymetrix microarray analyses.

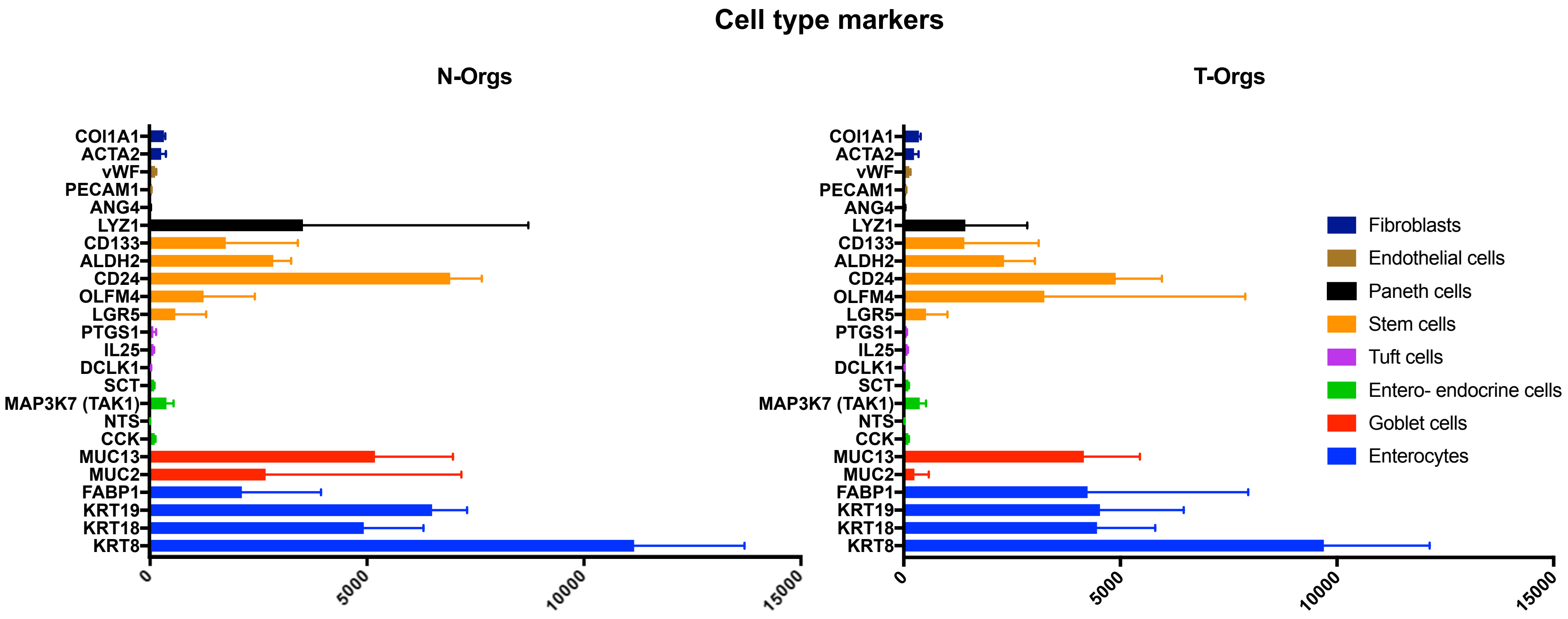

Suppl. Fig. 3: Expression levels of cell-type-markers in Tumor and Normal Organoids (T-Orgs and N-Orgs, respectively). Expression levels of the cell-type markers of the indicated cell types expressed in both N- and T-Orgs. Numbers on the X-axis are the average values of all patients (unlogged data) +/- SEM obtained by Affymetrix array analyses.

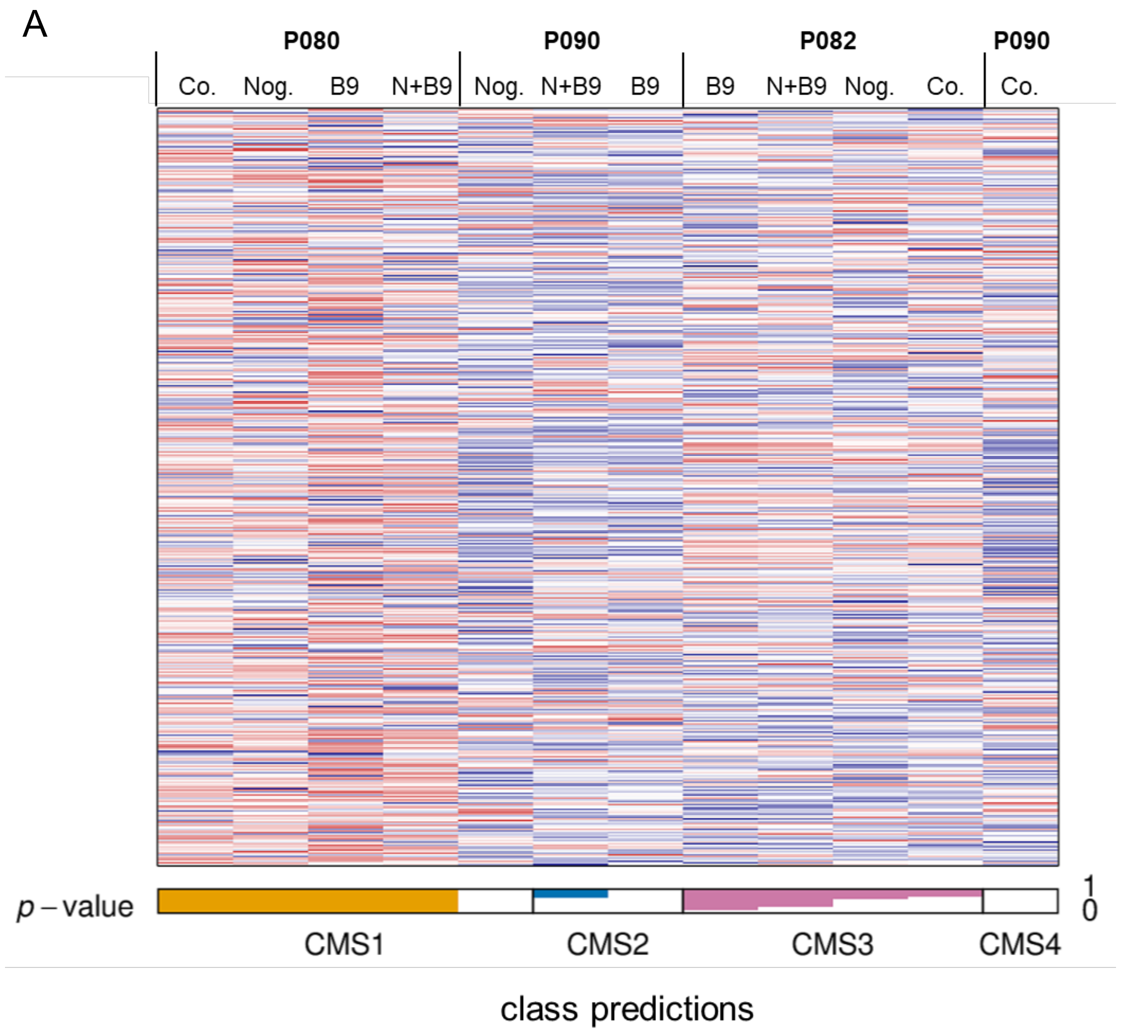
A
P080
P090
P082
P090
Co.
Nog.
B9
N+B9
Nog.
N+B9
B9
B9
N+B9
Nog.
Co.
Co.
p – value
1
0
CMS1
CMS2
CMS3
CMS4
class predictions

B

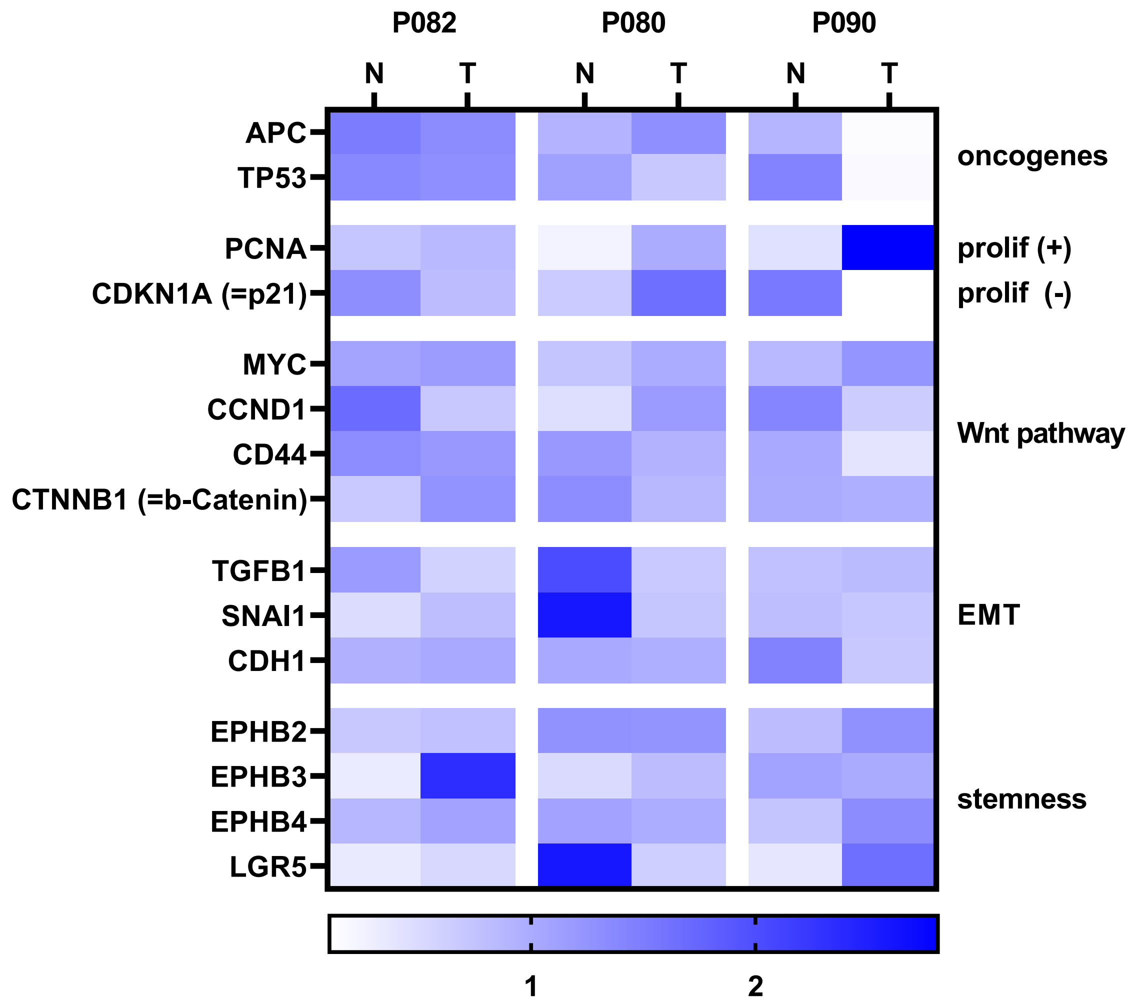

Suppl. Fig. 4: Analysis of the consensus molecular subtypes (CMS) of organoids from patients 082, 080 and 090. A) Analysis of the expression profiles of T-Orgs from all 3 patients (Co: untreated; Nog: noggin-treated; B9: BMP-9-treated; N+B9: treated with both, BMP-9 and noggin simultaneously). "CMScaller", an R package that was established in order to define the CMS of samples from colorectal cancer pre-clinical models, like organoids, based on expression profiles was used (Eide et al., 2017). With an input of only 12 samples many p-values did not reach statistical significance (white areas in the p-value bar at the bottom). Nevertheless the results imply that P080 belongs to CMS1, P082 to CMS3 and P090 either to CMS2 or 4. B) Heat-map showing the expression levels (unlogged values taken from affymetrix array results, normalized to average) of selections of marker genes in N- as well as corresponding T-Orgs of the 3 patients. Proliferation (+): pro-proliferative gene; Proliferation (-): anti-proliferative gene; EMT: epithelial to mesenchymal transition.

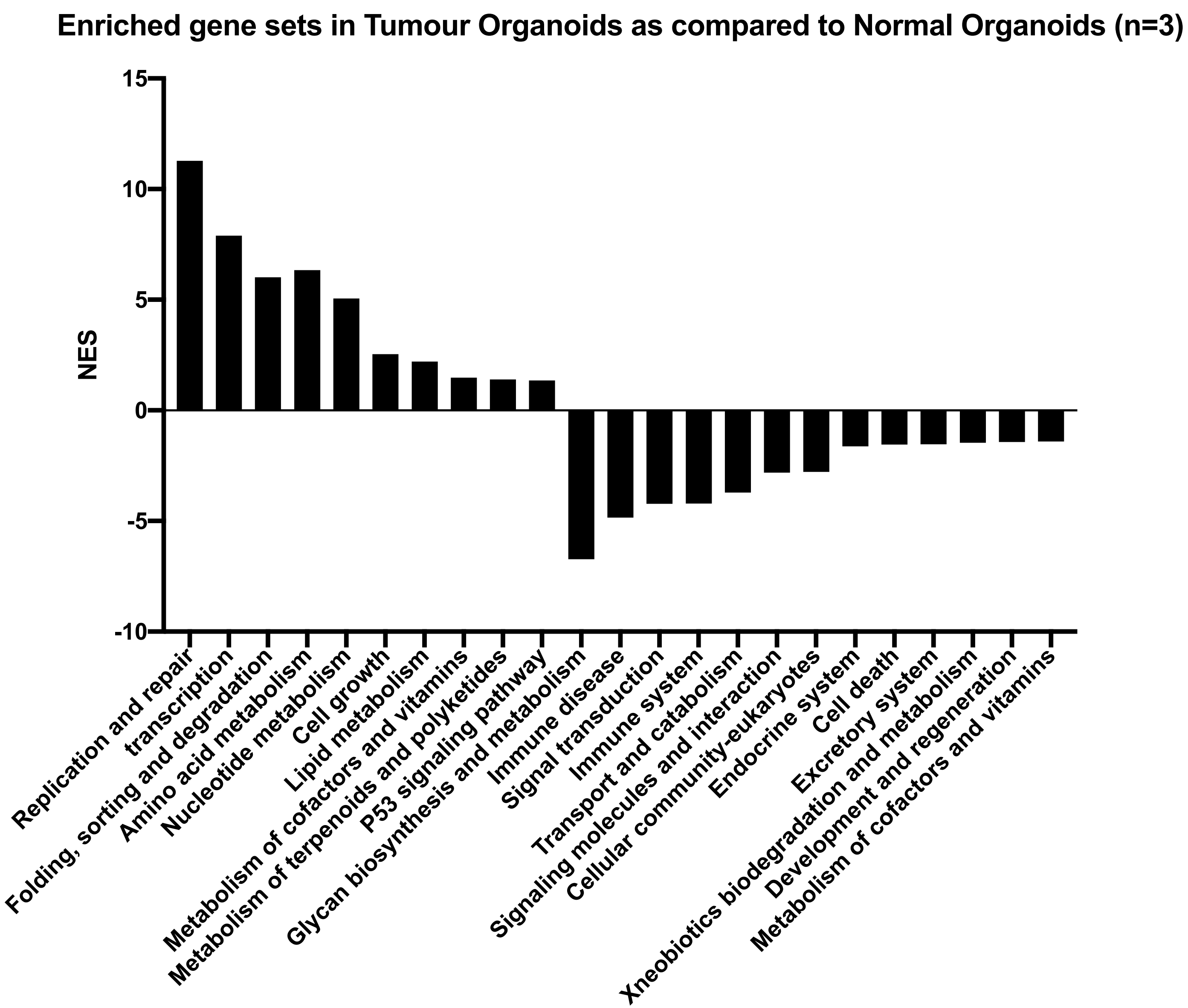

Suppl. Fig. 5: Enriched gene sets in T-Orgs compared to N-Orgs of all 3 patients. Gene sets enrichment analysis (GSEA) was performed focusing on the Kyoto Encyclopedia of Genes and Genomes (KEGG). A false discovery rate (FDR) lower than 0.25 and a p-value lower than 0.05 was considered as statistically significant. NES: normalized enrichment score.

A

top 30 down-regulated genes

sorted by patient 080

	Gene Symbol	P080 fold change	P082 fold change	P090 fold change
1	MT2A	-6,55	-0,26	0,58
2	MT1F	-5,11	-0,82	0,75
3	MAP1B	-5,02	-0,25	-0,53
4	KRT39	-4,88	0,32	0,48
5	OR51E1	-4,74	0,07	-0,30
6	GABRA3	-4,60	-0,26	-0,17
7	TMEM150C	-4,39	2,43	-0,23
8	MT1E	-4,31	-3,03	-0,42
9	NRCAM	-4,21	0,44	4,25
10	TNFRSF10C	-4,19	-3,73	-0,14
11	NFE2	-4,18	2,37	-1,26
12	RHOBTB1	-4,16	0,11	-0,38
13	SCD5	-4,16	-1,48	2,62
14	RGCC	-4,13	0,86	0,76
15	ABHD12B	-4,06	1,36	0,08
16	PCDH19	-4,01	-0,30	5,44
17	MT1B	-3,96	-0,67	-0,42
18	IGF2BP1	-3,95	-0,25	0,41
19	ROBO1	-3,90	0,91	-2,07
20	NRXN3	-3,83	2,49	1,78
21	PROM1	-3,67	-3,88	5,58
22	CADM1	-3,66	0,29	1,49
23	PHLDB2	-3,64	-0,12	-1,11
24	MT1A	-3,55	-0,23	0,31
25	TLE4	-3,50	-0,39	-1,21
26	MRPS21	-3,48	-0,44	0,17
27	FAM3B	-3,43	5,29	-1,73
28	TMEM200A	-3,37	-0,89	1,40
29	DMD	-3,25	-0,02	2,61
30	MEF2C	-3,22	-4,22	0,05

sorted by patient 082

Gene Symbol	P080 fold change	P082 fold change	P090 fold change
CA1	0,59	-8,25	0,81
CLCA4	-0,23	-7,11	-0,80
MMP7	3,70	-6,92	-0,70
REG4	4,53	-6,90	0,30
TCN1	0,10	-6,39	-0,10
CTSE	4,96	-6,19	-1,80
MUC2	2,53	-6,08	-0,23
PIGR	6,85	-6,06	-0,89
CA2	0,69	-5,60	-0,38
PLAC8	0,64	-5,36	0,18
SI	0,15	-5,20	-0,53
RARRES1	0,44	-5,18	0,92
F3	0,62	-5,09	-0,32
LYZ	0,34	-5,07	-0,92
NTRK2	0,24	-5,04	0,37
EVI2B	0,86	-5,03	0,76
GCNT3	3,74	-5,02	-2,05
SLC38A5	-0,54	-4,97	5,24
EPB41L4A	0,72	-4,86	-1,00
SLC6A14	1,27	-4,86	-1,36
DHRS9	2,08	-4,80	-0,29
SLC4A4	-0,29	-4,78	0,82
FAM198B	1,93	-4,78	-1,03
CLCA1	0,42	-4,73	0,05
IGFBP3	-0,08	-4,72	0,92
SPON1	-0,21	-4,68	0,33
AGR3	2,49	-4,53	0,66
PADI2	2,50	-4,39	-0,49
FER1L6	2,26	-4,38	0,00
LYPD8	-0,67	-4,36	1,25

sorted by patient 090

Gene Symbol	P080 fold change	P082 fold change	P090 fold change
HMGCS2	-0,67	1,02	-5,60
ID1	2,12	3,49	-5,59
CXCL5	-0,09	5,00	-5,52
FABP1	5,51	-1,09	-5,43
LCP1	-0,10	5,86	-5,08
TREML2	-2,56	2,70	-4,81
ADGRG7	-3,18	0,10	-4,63
FRMD3	1,35	-0,64	-4,56
ST3GAL4	0,34	-1,98	-4,48
TDRD1	0,65	4,04	-4,33
DMKN	0,73	-0,99	-4,30
OAS1	0,88	2,04	-4,20
PLOD2	0,07	0,69	-4,12
C10orf99	0,36	1,10	-4,09
CEACAM5	5,11	-0,54	-3,99
PGC	1,85	3,42	-3,97
FXYD3	1,48	-1,72	-3,84
SLCO1B3	-0,98	-2,22	-3,82
CDKN1A	1,21	-0,68	-3,82
ID3	3,42	2,91	-3,81
IFI27	3,10	-1,46	-3,75
LGALS1	2,15	2,54	-3,74
S100A4	2,90	1,77	-3,73
DAB2	1,44	-1,32	-3,72
CYP2B6	-0,28	2,96	-3,63
CDA	2,00	0,78	-3,60
PADI3	0,71	1,27	-3,59
COL17A1	1,02	-3,04	-3,57
FER	0,42	1,57	-3,56
KRT20	1,62	-0,96	-3,52

B

top 30 up-regulated genes

sorted by patient 080

	Gene Symbol	P080	P082	P090
		fold change		
30	CDK1	3,79	1,38	0,26
29	CCNA2	3,88	0,01	1,77
28	TOP2A	3,90	1,56	1,27
27	ARHGAP11A	3,92	1,49	1,28
26	RRM2	3,92	1,06	1,19
25	ATOH1	3,94	-2,17	0,36
24	DPP4	4,02	-1,67	-2,41
23	CEACAM7	4,02	-3,63	-1,79
22	PCLAF	4,09	1,19	1,62
21	DLGAP5	4,19	0,43	1,10
20	ACAT2	4,22	0,82	0,39
19	TSPAN7	4,27	-0,12	0,09
18	FADS2	4,31	-3,48	-1,58
17	AKR1B10	4,38	-2,87	-1,44
16	IL33	4,42	0,92	-3,20
15	EREG	4,45	1,42	0,87
14	REG4	4,53	-6,90	0,30
13	SRPX	4,53	0,12	1,09
12	SYTL5	4,53	-1,39	0,01
11	SLC9A2	4,64	-0,30	-2,21
10	SERPINA1	4,89	-0,10	-1,53
9	CEACAM6	4,93	0,44	-3,38
8	C1orf21	4,96	-1,33	1,86
7	CTSE	4,96	-6,19	-1,80
6	IL2RG	4,96	-1,34	-0,62
5	TRIM31	4,96	-1,66	-3,05
4	ANPEP	5,06	-4,21	0,11
3	CEACAM5	5,11	-0,54	-3,99
2	FABP1	5,51	-1,09	-5,43
1	PIGR	6,85	-6,06	-0,89
43	**PTPRO**	3,62	4,72	3,59

sorted by patient 082

Gene Symbol	P080	P082	P090
		fold change	
FABP6	1,56	3,21	-1,85
PTCH1	-0,96	3,28	-2,99
NCAM1	-0,04	3,31	-2,81
RUBCNL	-1,88	3,32	0,07
GNG4	-1,43	3,39	0,83
PGC	1,85	3,42	-3,97
KRT23	-1,48	3,45	-0,19
ID1	2,12	3,49	-5,59
CRNDE	0,30	3,50	1,43
MAN1A1	-0,49	3,51	0,64
FBXO27	-1,10	3,56	-1,49
GLIPR1	-0,54	3,59	-1,79
HUNK	-0,30	3,60	0,51
EYA1	-0,02	3,63	-1,98
LRP4	-2,40	3,76	0,80
IFITM1	-1,04	3,76	-3,37
PTPRD	-1,17	3,79	1,15
BCL11A	-1,17	3,99	1,03
KRT75	-0,25	4,04	-2,88
TDRD1	0,65	4,04	-4,33
PAH	0,42	4,10	-1,69
APCDD1	-1,88	4,12	-1,17
AGMO	-0,52	4,34	-3,06
DACH1	-1,29	4,47	1,85
PROX1	-0,83	4,50	1,00
**PTPRO**	3,62	4,72	3,59
CXCL5	-0,09	5,00	-5,52
FAM3B	-3,43	5,29	-1,73
LCP1	-0,10	5,86	-5,08
PCP4	-2,91	6,40	-0,71

sorted by patient 090

Gene Symbol	P080	P082	P090
		fold change	
PRNP	0,21	-2,05	2,88
SLC6A20	1,91	-1,67	2,89
GNPDA2	0,21	-1,97	2,92
SLFN13	1,53	-0,83	3,00
RNF182	-0,23	-0,32	3,06
CLDN2	-1,90	-1,46	3,09
OXCT1	1,83	-1,13	3,20
DRD2	-0,45	0,56	3,25
ZNF22	-0,42	-0,75	3,36
GSTM3	1,52	-3,91	3,41
DPP10	-0,12	-0,55	3,47
SLAMF6	0,40	0,41	3,48
NECTIN3	-0,17	-1,60	3,53
C3orf14	2,70	-0,60	3,58
**PTPRO**	3,62	4,72	3,59
GABRB2	-0,35	0,22	3,73
REG1A	1,17	-0,10	3,87
SPTLC3	-0,67	-1,68	4,13
CPA2	-2,39	0,36	4,14
ZNF286A	1,46	-3,25	4,14
NRCAM	-4,21	0,44	4,25
CST7	-2,55	0,06	4,34
HEXA	-0,20	-3,11	4,36
CYP4X1	-2,35	-0,71	4,50
SLC38A5	-0,54	-4,97	5,24
ANOS1	0,35	-0,45	5,24
UCHL1	0,39	-1,95	5,37
PCDH19	-4,01	-0,30	5,44
PROM1	-3,67	-3,88	5,58
OLFM4	0,22	1,58	6,22

Suppl. Fig. 6: Individual expression changes in T-Orgs compared to corresponding N-Orgs. Genes down-regulated (A) and up-regulated (B) in T-Orgs vs. N-Orgs of the three patients (P080, P082 and P090).

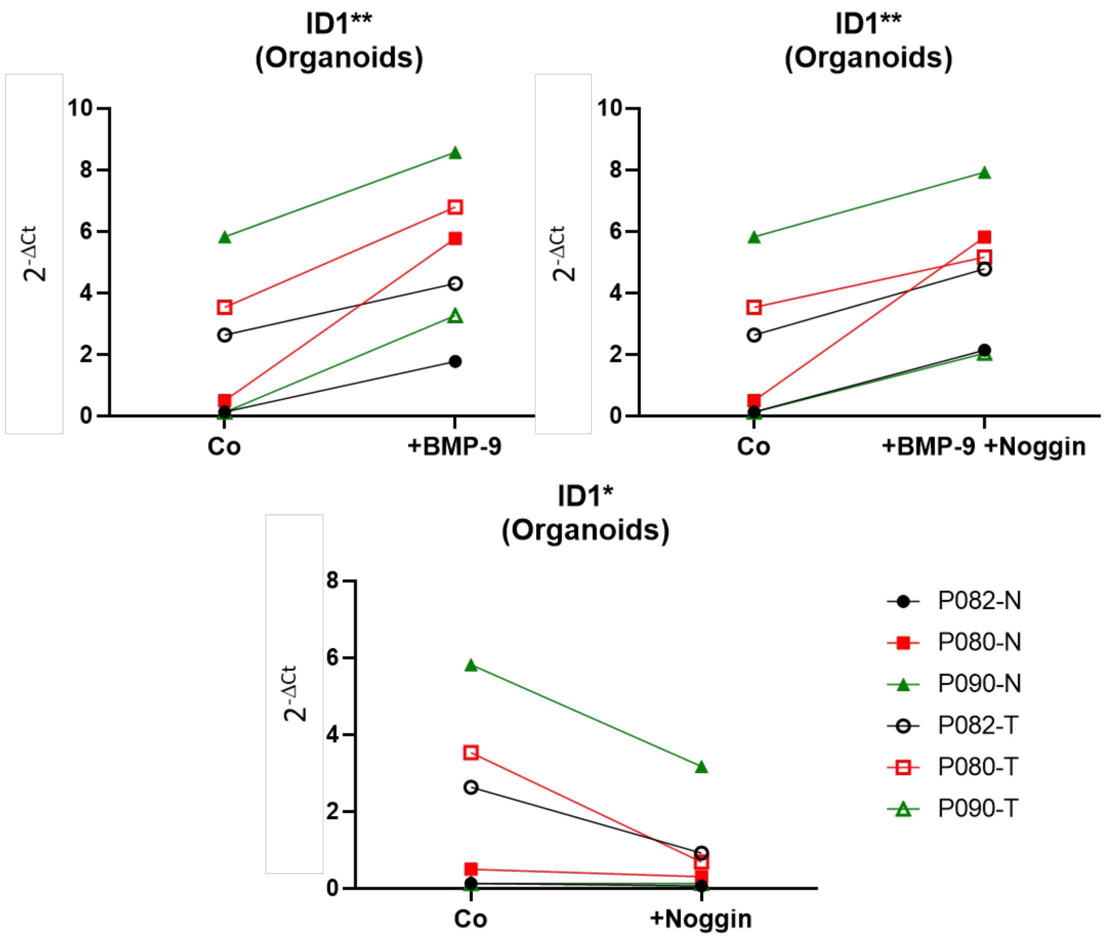

Suppl. Fig. 7: Noggin reduces and BMP-9 enhances ID1 expression in Organoids. As validation of the array data shown in Fig. 5A real-time PCR was performed using the same samples. Organoids were derived from human biopsies of normal and cancerous gut mucosa as depicted in suppl. fig.2 followed by in vitro stimulation with either recombinant Noggin (100 ng/ml) or BMP-9 (5 ng/ml) or both together. After 48 h RNA was isolated and processed for real-time PCR analyses. The scores (normalized to the house-keeping gene rS18) for the individual ID1 expression in each sample (N as well as T of each patients organoids) are plotted and values of untreated (Co.) are compared to either BMP-9-treated (+BMP-9), Noggin-treated (+Noggin) or treated with both (+BMP-9 +Noggin). For statistics all 6 controls were compared to all 6 treated samples and significance was calculated using the paired t-test. *=p>0.05; **=p>0.01.

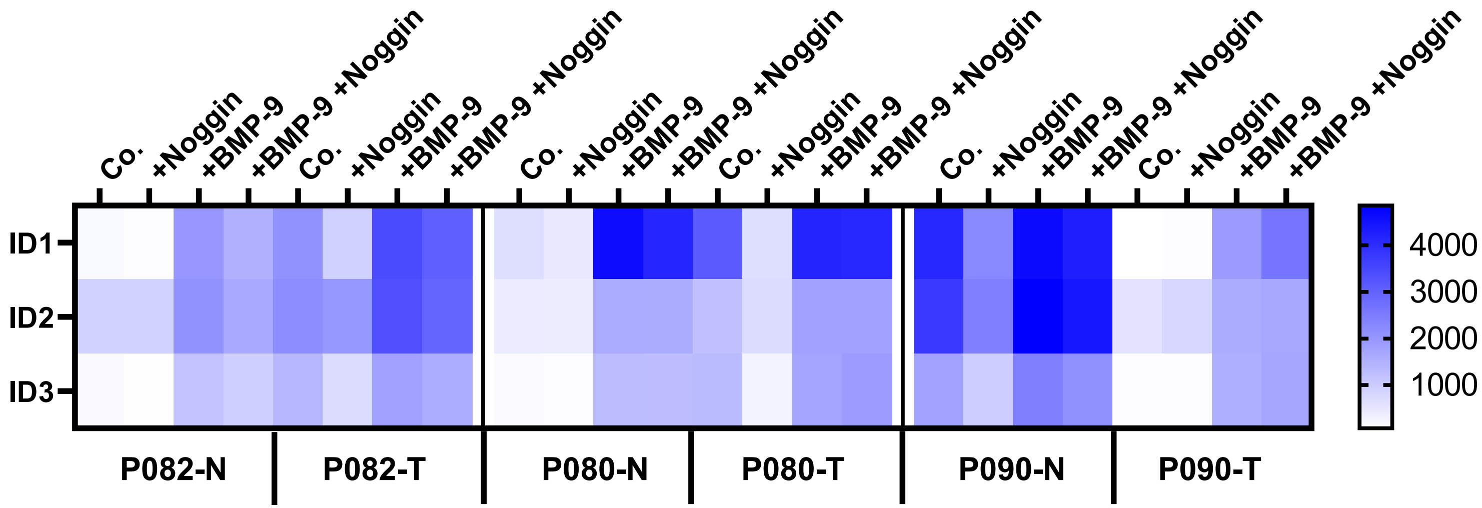

Suppl. Fig. 8: Heat-map showing the individual expression levels (unlogged values taken from affymetrix array results) of ID1, 2 and 3 in N- as well as corresponding T-Orgs of the 3 patients.

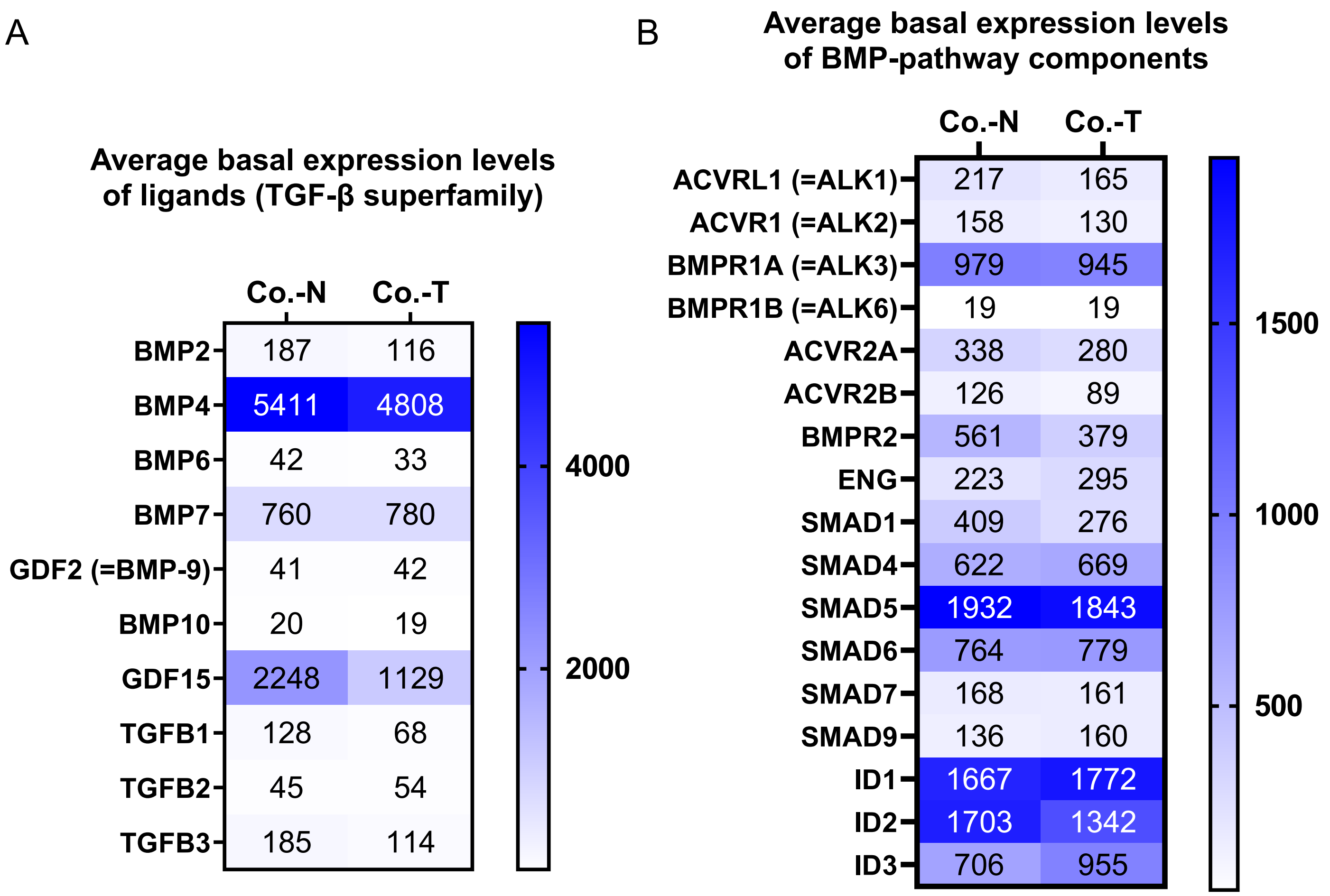

Suppl. Fig. 9: Heat-map showing the average expression levels (unlogged values taken from affymetrix array results) of (A) a selection of ligands of the TGF-β superfamily and (B) a selection of components of the BMP-pathways as well as the target genes ID1-3 in N- compared to T-Orgs. Note that none of the average changes from N- to T-Orgs were statistically significant.
